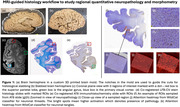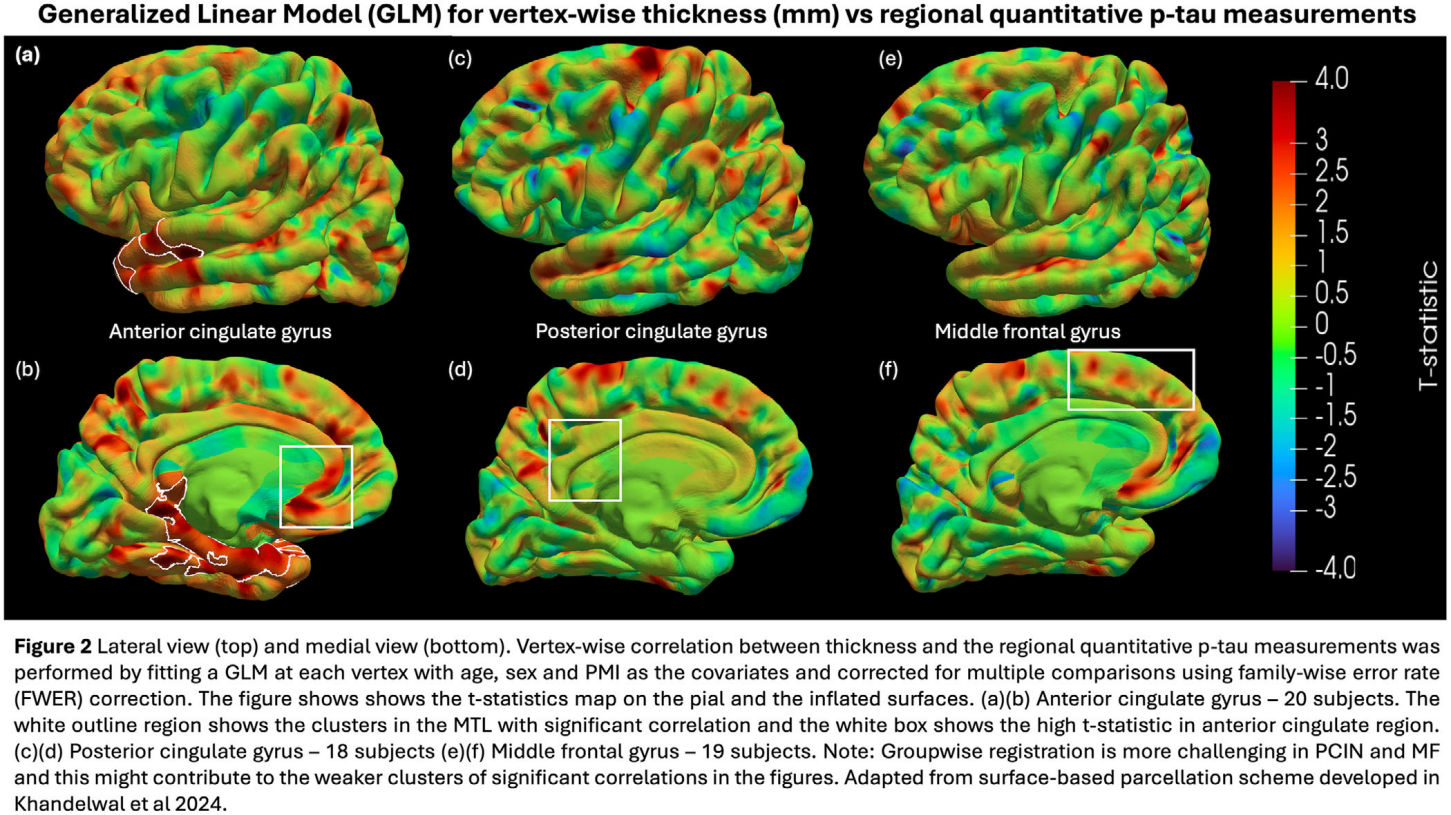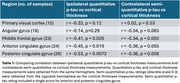# Operationalizing postmortem pathology‐MRI association studies in ADRD with MRI‐guided histology sampling: does closer proximity lead to stronger associations?

**DOI:** 10.1002/alz.095711

**Published:** 2025-01-09

**Authors:** Chinmayee Athalye, Pulkit Khandelwal, Alejandra Bahena, Winifred Trotman, Daniel T Ohm, Sheina Emrani, Eric Teunissen‐Bermeo, Noah Capp, Sydney A Lim, Lisa M Levorse, Amanda E Denning, Ranjit Ittyerah, Theresa Schuck, Marianna Gabrielyan, Karthik Prabhakaran, Gabor Mizsei, John L. Robinson, John A. Detre, Eddie B Lee, David J Irwin, Corey T McMillan, M. Dylan Tisdall, Sandhitsu R. Das, David A Wolk, Paul A. Yushkevich

**Affiliations:** ^1^ University of Pennsylvania, Philadelphia, PA USA

## Abstract

**Background:**

Histopathological analysis of autopsied brains is the gold standard of diagnosis in neurodegenerative disorders. Co‐registered histology and MRI scans aid in understanding pathology and structural features. Previous studies focused on the medial temporal lobe (MTL) for atrophy patterns in phosphorylated tau (p‐tau) pathology and in whole hemisphere scans with contralateral semi‐quantitative p‐tau measures. Here, we extend this to study ipsilateral quantitative p‐tau and cortical thickness.

**Method:**

We used 29 brain hemispheres from patients with Alzheimer’s Disease (AD) diagnosis. We identified 19 regions of interest (ROIs), spanning the cortex and MTL. The ROIs were manually marked on the T2‐weighted 7T hemisphere MRI (0.3mm3 isotropic). The MRI scans were used to build a 3D‐printed mold of the brain. This mold helped identify the slabs with ROIs for histological staining. We used LFB‐CV (Luxol fast blue cresyl violet) stain and AT‐8 antibody for p‐tau on each ROI slide, which were then registered to the whole hemisphere MRI scans in a semi‐automatic manner. We used a weakly‐supervised learning algorithm WildCat to get quantitative measures of p‐tau for each ROI in the histology. Regional cortical thickness at the ROI markers was measured as the diameter of the maximally inscribed sphere in the topologically‐corrected automated deep learning‐based segmentations. Separately, whole hemisphere‐based thickness measurements were derived using a surface‐based parcellation scheme.

**Result:**

One‐sided Spearman rank test was performed to assess the correlation between p‐tau and cortical thickness measurements. For ipsilateral quantitative p‐tau, this test showed significant correlation in the posterior cingulate gyrus (r = ‐0.53, p‐value = 0.0072), anterior cingulate gyrus (r = ‐0.45, p‐value = 0.019), and the middle frontal gyrus (r = ‐0.41, p‐value = 0.025). We performed the same test with contralateral semi‐quantitative tau scores (Table 1). A generalized linear model showed significant correlations between whole hemisphere vertex‐wise thickness with regional measurements of neuronal tangles in the anterior cingulate (Figure 2).

**Conclusion:**

Postmortem MRI‐guided ipsilateral quantitative p‐tau burden measures showed better correlation with cortical thickness, in absolute terms, than contralateral semi‐quantitative tau scores. Replication and understanding whether co‐localization or improved quantification are most responsible for this improvement will require larger brain donor cohorts.